# Effects of Dihydroartemisinin and Artemether on the Growth, Chlorophyll Fluorescence, and Extracellular Alkaline Phosphatase Activity of the Cyanobacterium *Microcystis aeruginosa*

**DOI:** 10.1371/journal.pone.0164842

**Published:** 2016-10-18

**Authors:** Shoubing Wang, Ziran Xu

**Affiliations:** Department of Environmental Science and Engineering, Fudan University, Shanghai, People’s Republic of China; VIT University, INDIA

## Abstract

Increased eutrophication in the recent years has resulted in considerable research focus on identification of methods for preventing cyanobacterial blooms that are rapid and efficient. The objectives of this study were to investigate the effects of dihydroartemisinin and artemether on the growth of *Microcystis aeruginosa* and to elucidate its mode of action. Variations in cell density, chlorophyll a, soluble protein, malondialdehyde, extracellular alkaline phosphatase activity (APA), and chlorophyll fluorescence parameters (Fv/Fm, ΦPSII, ETR, rapid light curves, fast chlorophyll fluorescence curves on fluorescence intensity, and relative variable fluorescence) were evaluated by lab-cultured experiments. Our results demonstrated that both dihydroartemisinin and artemether inhibited the growth of *M*.*aeruginosa* by impairing the photosynthetic center in photosystem II and reducing extracellular APA, with a higher sensitivity exhibited toward artemether. The inhibitory effects of dihydroartemisinin on *M*.*aeruginosa* increased with concentration, and the maximum growth inhibitory rate was 42.17% at 24 mg·L^-1^ after 120h exposure, whereas it was 55.72% at 6 mg·L^-1^ artemetherafter 120h exposure. Moreover, the chlorophyll fluorescence was significantly inhibited (p<0.05) after 120h exposure to 12 and 24 mg·L^-1^ dihydroartemisinin. Furthermore, after 120h exposure to 6 mg·L^-1^ artemether, Fv/Fm, ΦPSII, ETR and rETR_max_ showed a significant decrease (p<0.01) from initial values of 0.490, 0.516, 17.333, and 104.800, respectively, to 0. One-way analysis of variance showed that 6 mg·L^-1^ artemether and 24 mg·L^-1^ dihydroartemisinin had significant inhibitory effects on extracellular APA (p<0.01). The results of this study would be useful to further studies to validate the feasibility of dihydroartemisinin and artemether treatment to inhibit overall cyanobacterial growth in water bodies, before this can be put into practice.

## Introduction

Worldwide, harmful algal blooms (HABs) of freshwater aquatic ecosystems, especially *Microcystis aeruginosa* (*M*. *aeruginosa*) blooms, have resulted in the reduction of biodiversity, ecosystem functions, and deterioration of surface water quality [1]. HABs are responsible for the production of a wide variety of potent toxins that play a role in fish kills, high turbidity, foul smells, serious economic losses, human illnesses, and can even cause death [2]. As a result, HAB control measures have become one of the key topics in eutrophic-related studies in recent times [3].

Currently, several approaches have been proposed and studied for the removal of HABs. These methods include mechanical, physical (ultrasonication, coagulation/flocculation and centrifugal separation), biological (Traditional biomanipulation and Non-Traditional biomanipulation), and chemical (oxidants and metal-ion compounds) methods [4–5]. Although these approaches can efficiently remove cyanobacteria from polluted surface water, most of them have operational, economic, and environmental limitations [6]. Taking these limitations into account, it is necessary to develop a new generation of algal inhibitors to only target HAB communities. These new inhibitors will still have to be effective, applicable, economical, and environmentally benign.

Several studies have confirmed that the discovery of anti-algal allelochemicals, extracted from autotrophs, might provide potential cyanobacterial growth inhibitors to control HABs [6–8]. The production and excretion of allelochemicals from natural macrophytes and advanced plants is considered an environmentally friendly strategy to control cyanobacterial blooms. However, to our knowledge, few studies have focused on the derivatives of allelochemical algaecides isolated from natural organisms. Previous studies have demonstrated that the allelochemical substance artemisinin, isolated from the compositae plant *Artemisia annua*, has already been confirmed to possess strong inhibition effects on *M*. *aeruginosa* [9–12]. Otherwise, it has been found that the ethyl acetate and petroleum ether extracts from *Artemisia annua* exhibited the strong inhibition activity on *M*. *aeruginosa* [13].

Nevertheless, the feasibility of applying artemisinin and its derivatives, petroleum ether and ethyl acetate extract, to inhibit overall cyanobacterial growth in water bodies is not clearly understood. Application in natural systems poses many problems, such as dilution effects, doses, and effects on non-target organisms. Despite this, more effective and practical allelochemical drugs need to be explored. Further, studying the mechanism of algistatic effects of these drugs could benefit their real application. However, before any new drugs can be utilized in natural systems, more detailed research needs to be carried out.

As there is no current research focusing on the antibacterial or antialgal activity of dihydroartemisinin and artemether derived from artemisinin, a primary investigation is conducted on the effect of dihydroartemisinin and artemether on the growth of *M*. *aeruginosa* in our lab.The aim of our study was to examine the feasibility of using dihydroartemisinin and artemether to control algal blooms and to elicit the physiological responses of cyanobacteria to artemisinin derivatives produced by autotrophs.

## Material and Methods

### Algae culture

*M*.*aeruginosa* (FACHB-930) was purchased from the Freshwater Algae Culture Collection, Institute of Hydrobiology, Chinese Academy of Sciences (Wuhan, China) and cultivated using BG11 medium in 1-L autoclaved conical flasks. The reactors were placed in an incubator at a controlled temperature of 25 ± 1°C. The flasks were illuminated by an irradiance of 58 μmol photons·m^-2^·s^-1^ by tubular fluorescent lamps with a 12-h light-dark cycle, and were shaken four times daily. In this study, cells in the exponential growth phase were used, as determined by measuring the cell density every day. All the reagents and solvents were of at least analytical grade except as noted. All the flasks and the culture medium were sterilized at 121°C for 30 min.

### Algicidal assays

*M*. *aeruginosa* cells in the logarithmic growth phase were harvested and cultured in 350-mL flasks with 300 mL BG11 medium. Various doses of dihydroartemisinin and artemether (obtained from Aladdin biochemical science and Technology Co., Ltd.) were added to the cultures at concentrations of 6, 12, and 24 mg·L^-1^. The choice of concentrations, comparable with other natural allelochemicals, was based on previous pilot experiments and investigations for physiological effects on *M*. *aeruginosa* by a range of artemisinin concentrations [9–12]. Controls were prepared by inoculating *M*. *aeruginosa*into culture medium without the addition of dihydroartemisinin and artemether. Three replicates were made in the experiment and all the operations were carried out in axenic conditions. Cell density measurements were carried out at 0, 6, 12, 24, 48, 72, 96, and 120 h after the onset of different treatments. Chlorophyll a, soluble protein, and alkaline phosphatase activity (APA) assays were performed at 0, 24, 48, 72, 96, and 120h after the onset of different treatments. The determination of malondialdehyde was taken after 120-h incubation. In chlorophyll fluorescence parameters, the maximum effective quantum yield of photosystem II (PSII) (Fv/Fm), effective quantum yield of PSII (ΦPSII), and electron transport rate (ETR) were monitored when *M*. *aeruginosa* was incubated with dihydroartemisinin and artemether at 6, 24, 48, 72, 96, and 120h. The fast chlorophyll fluorescence curves (fluorescence intensity vs. time and relative variable fluorescence vs. time) at 72, 120h and rapid light curves (relative electron transport rate vs. intensities of actinic light) were produced at 6, 72, and 120h.

### Measurements of cell density and Chlorophyll a (Chl-a)

Cell density was determined by measuring the optical density at 680 nm (OD_680_) with a visible spectrophotometer (752N; Shanghai Lengguang Industrial Co., Ltd., Shanghai, China) to represent relative cyanobacterial biomass [14]. Cell numbers were determined by a microscope using the hemocytometer counting method. The regression equation between OD_680_ (Y) and the cell density (X, ×10^6^ cell/mL) was established as Y = 0.056X−0.005 (R^2^ = 0.99). The inhibition ratio (IR) was used for expressing the effects of algaecide, which was calculated as follows:

$$\text{IR}\left(\text{\%}\right)=\left(1-\frac{\text{T}}{\text{C}}\right)\times 100\text{\%}$$ (1)

T and C: cell density of treatment and control

The measurement of Chl-*a* followed the method described by Wang et al. [6]. Briefly, 3-mL samples were filtered through a 0.45-μm Millipore filter and then extracted with 3 mL of 90% acetone at 4°C for 24 h. The extract was then centrifuged and the Chl-*a* content was measured with a spectrophotometer at wavelengths of 750, 663, 645, and 630nm. Chl-*a* content was calculated as follows:

$$\text{Chl-}a(\mu \text{g}\cdot {\text{L}}^{-1})=\frac{(11.64{\times \text{A}}_{663}{-\text{A}}_{750})-2.16\times \left({\text{A}}_{645}{-\text{A}}_{750}\right)\text{+0.10}\times \left({\text{A}}_{630}{-\text{A}}_{750}\right))\times \text{V1}}{\text{V}}$$ (2)

where A_750_, A_663_, A_645_, and A_630_ are the absorbance of the mixtures at 750, 663, 645, and 630nm, respectively; V1: volume of 90% acetone (mL), V: volume of water sample (L).

### Determination of soluble protein and malondialdehyde (MDA)

Coomassie brilliant blue G-250 method was used to detect the soluble protein content [15]. Bovine serum albumin was used as the standard. After 120h incubation, *M*. *aeruginosa* cells were filtered through a 0.45-μm Millipore filter. After cutting, filters were extracted with 10% trichloroacetic acid (TCA) and the mixture was subsequently centrifuged at 4000rpm for 20 min. A mixture of the supernatant and equal volume 0.67% thiobarbituric acid (dissolved in 10% TCA) was heated in a 100°C water bath for 30 minand then cooled down immediately to measure the absorbance at 600, 532, and 450nm. The MDA content was calculated as follows [16]:

$$\text{C}=\frac{\left[6.45\times \left({\text{A}}_{532}-{\text{A}}_{600}\right)-0.56\times {\text{A}}_{450}\right]\times \left(\text{V}+{\text{V}}_{\text{e}}\right)\times \frac{{\text{V}}_{\text{t}}}{{\text{V}}_{\text{e}}}}{1000\times {\text{V}}_{\text{a}}\times {\text{S}}_{\text{a}}}$$ (3)

where A_450_, A_532_, and A_600_ are the absorbance of the mixtures at 450, 532, and 600nm, respectively; C is the content of MDA (mmol/cell); V is volume (L) of thiobarbituric acid; V_t_ and V_e_ are the total and the extract volume of TCA (L); V_a_ and S_a_ are the volume (mL) and cell density (cell/mL) of *M*. *aeruginosa*.

### Determination of chlorophyll fluorescence parameters

The chlorophyll fluorescence parameters were measured once the sample had been stirred and dark-adapted for 15 min in a PAM cuvette (Water-PAM, Heinz Walz GmbH, Effeltrich, Germany). Measurements were taken using a Water-Pulse-Amplitude-modulated fluorescence monitoring system. The photochemical energy conversion of photosystem II is zero when the entire reaction center of this system is in a fully closed sate and all of the non-photochemical processes occur in minimum time to get the Fm value. This study utilized the most representative photosynthetic parameters for photosystem II (Fv/Fm, ΦPSII and ETR). Additionally, rapid light curves (RLCs) were determined, based on measurements of relative electron transport rates (rETR). rETR values were collected from samples exposed to 10 intensities of actinic light ranging from 0 to 1295 μmol photons·m^−2^·s^−1^ obtained from the Water-PAM rapid light curves. Fast chlorophyll fluorescence curves, based on measurements of fluorescent intensity, were determined and the relative variable fluorescence was calculated using the equation: Vt = (Ft−Fo)/(Fm−Fo). Otherwise, this study used an empirical function to estimate RLCs and rETR [17]. The rETR of PSII could be calculated by the equation:

rETR=(Fm'−Fs)/Fm'×0.84×0.5×PAR (4)

where 0.84 is the proportion of incident light absorbed by the algae, of which approximately 50% is transferred to PSII [18]; PAR is light irradiances (μmol photons·m^−2^·s^−1^); Fm' is the maximal fluorescence in the light-adapted state; Fs is the steady-state fluorescence yield.

### Extracellular Alkaline phosphatase activity (APA) measurements

Extracellular APA was routinely assayed using the colorimetric method involving the hydrolysis of p-nitrophenylphosphate disodium hexahydrate (p-NPP, Sigma) to p-nitrophenol (PNP), by reading the absorbance at 410nm. The rate of PNP production was used as an indicator of APA. Two-milliliter samples were placed into a cuvette containing 1 mL Tris-HCl buffer (pH 8.4) and 2 mL p-nitrophenyl phosphate (pNPP). After incubation (6 hours at 30°C), the extracellular APA of samples were analyzed at 410nm spectrophotometrically. The measurement procedure followed the method described by Berman [19]. Controls, containing no substrate and no cells, were included to correct the absorbance changes due to cell density and spontaneous hydrolysis of the p-NPP.

### Statistical analysis

The results were considered significantly different means only if the probability (p) was < 0.05 and 0.01 by one-way analysis of variance (ANOVA). Data shown in this study are presented in means ± standard deviation (SD). All statistical analyses were conducted using SPSS 19.0. All figures were plotted using Origin 9.0.

## Results

### Inhibition effects of dihydroartemisinin and artemether on *M*. *aeruginosa* growth

**Fig 1:**
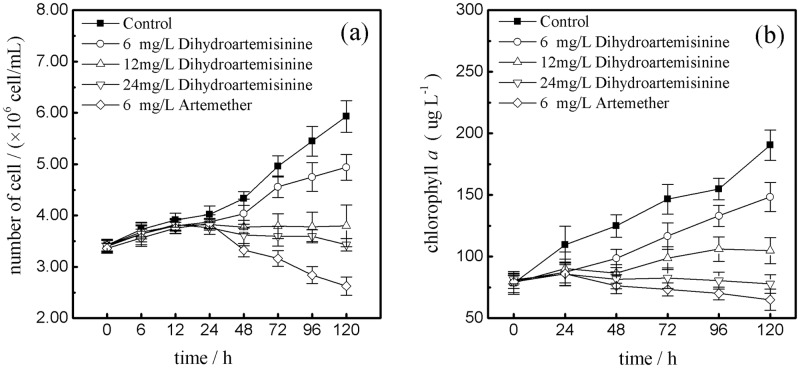
Effects of dihydroartemisinin and artemether on the cell density and chlorophyll a content of *M*. *aeruginosa*. ((a): cell density; (b): chlorophyll a).

The changes of *M*. *aeruginosa* cell density in each concentration groups are shown in Fig 1(a). The cell number of the control increased substantially from 3.42 to 5.93× 10^6^ cells mL^−1^, implying that cells were in the exponential growth phase during the experiment. Concretely, after 24 h growth of *M*. *aeruginosa* became inhibited after exposure to dihydroartemisinin and artemether. The inhibition rate reached its peak after approximately 120h with exposure to 6 mg·L^−1^ dihydroartemisinin (16.67%), 12 mg·L^−1^ dihydroartemisinin (35.94%), 24 mg·L^−1^dihydroartemisinin (42.17%) and 6 mg·L^-1^ artemether (55.72%). At the 6 mg·L^-1^, 24h after the start of artemether treatment, the cell density was inhibited significantly and with treatment time, the inhibitory affects became stronger. This would suggest that exposure time is a significant factor and that low concentrations artemether can sustain their inhibitory effects on *M*. *Aeruginosa* growth over a relatively long time. The trends in Chl-a content under different doses of dihydroartemisinin and artemether were shown in Fig 1(b). Results from the present investigation showed the trends in Chl-a content under various doses of dihydroartemisinin and artemether followed asimilar pattern to cell density. Furthermore, when the dihydroartemisinin concentrations were 6 and 12 mg·L^-1^, after treatment for 120h, the chl-a contents were significantly inhibited (p<0.01) compared to the control, but still increased slightly from 80.35 to 148.36 μg·L^-1^, and 78.67 to 104.85 μg·L^-1^, respectively. Meanwhile, the chl-a contentin 6 mg·L^-1^ artemether and 24 mg·L^-1^ dihydroartemisinin treatments significantly decreased (p<0.01) from 79.33 to 64.95 μg·L^−1^ and from 81.23 to 77.71 μg·L^-1^, respectively.

### Influence of Dihydroartemisinin and Artemether on the content of soluble protein and MDA

**Fig 2:**
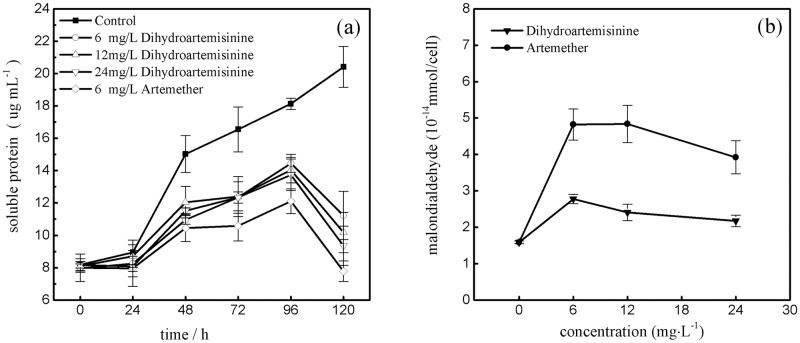
Effects of dihydroartemisinin and artemether on the content of soluble protein and 120h malondialdehyde in *M*. *aeruginosa* ((a): soluble protein; (b): malondialdehyde).

The effects of dihydroartemisinin and artemether on the soluble protein content at 0, 24, 48, 72, 96, and 120h exposure are shown in Fig 2(a). In the 96-h cultivation, the soluble protein content of dihydroartemisinin and artemether groups were generally significantly inhibited compared with controls, but increased slightly with treatment time. There were also considerable decreases in soluble protein content in all treatments at 120h. However, there were no different variation patterns in soluble protein content between dihydroartemisinin and artemether treatments. Over the total exposure time, the inhibition effects of artemether on soluble protein content were stronger than those of dihydroartemisinin. In our experiment, as shown in Fig 2(b), the MDA content of the control was 1.58 × 10^−14^ mmol/cell, which was lower than that of all treatments. The MDA content in algae exposed to artemether treatments were significantly higher than dihydroartemisinin treatments and the control. In addition, the MDA content showed a slight downward trend with dihydroartemisinin and artemether concentration, meaning the damage of antioxidant defense system on *M*. *aeruginosa* in higher concentrations.

### Change inchlorophyll fluorescence parameters

**Fig 3:**
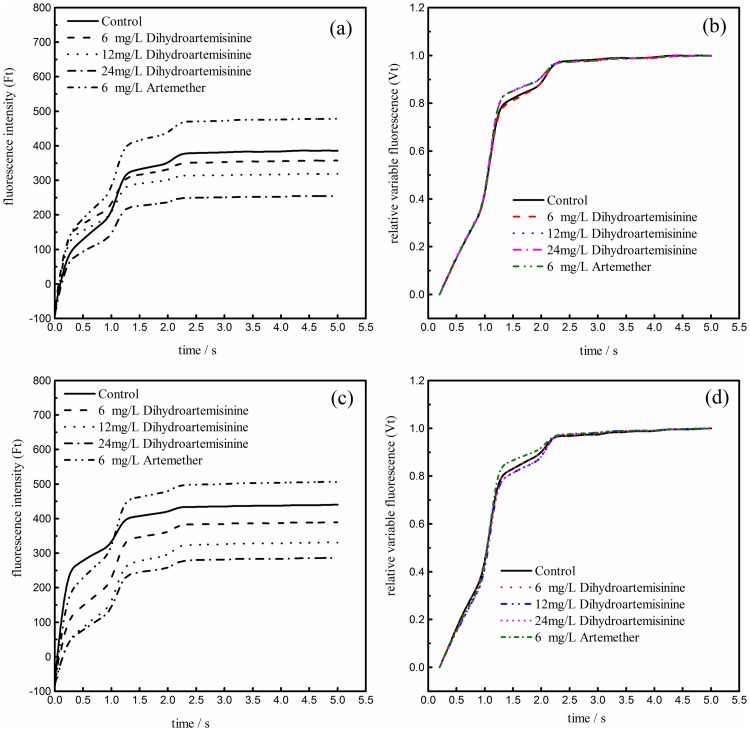
The fast chlorophyll fluorescence curves of *M*. *aeruginosa* after 72 and 120 h treatment with dihydroartemisinin and artemether at different concentrations. Ft represents fluorescence intensity at time t; Vt represents the relative variable fluorescence at time t, Vt = (Ft—Fo)/(Fm—Fo). ((a): 72h Ft; (b): 72h Vt; (c): 120h Ft; (d): 120h Vt).

The effects of different concentrations of dihydroartemisinin and artemether on the function of PSII in *M*. *aeruginosa* after 72 and 120h treatment, using fast chlorophyll fluorescence induction tests, were investigated. The fluorescence intensity and relative variable fluorescence variation curves at 72 and 120h are shown in Fig 3. As seen in Fig 3(a) and 3(c), the fluorescence intensity of all treatments and control at 72 and 120h rapidly increased to the maximum in less than 2.5 s and then gradually leveled off with increasing exposure time in both cases. The maximum fluorescence intensity decreased with the increasing dihydroartemisinin concentration. Similarly, the relative variable fluorescence of all groups at 72 and 120h rapidly increased from 0 to 1 in less than 2.5 s and then remained stable (Fig 3(b) and 3(d)). The trends of relative variable fluorescence in all treatments and control group at 72 and 120h are highly coincident, with an exception at the point where each turns before leveling off.

**Fig 4:**
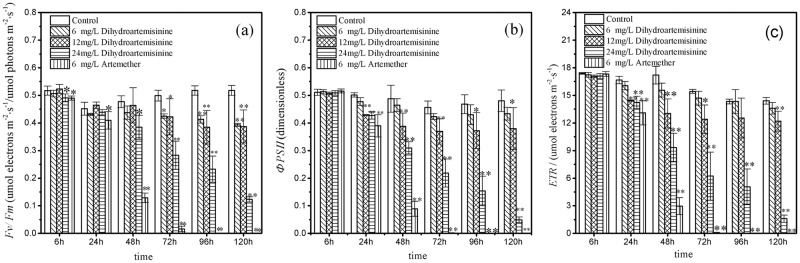
Chlorophyll fluorescence parameters of *M*. *aeruginosa* exposed to different dihydroartemisinin and artemether concentrations. (a: The maximum effective quantum yield; b: the effective quantum yield; c: the electron transport rate). *P<0.05; **P<0.01. Statistically significant differences when compared to the control without dihydroartemisinin and artemether.

The effects of dihydroartemisinin and artemether on Fv/Fm, YII, and ETR within the treatment time are shown in Fig 4. Both of the Fv/Fm, YII, and ETR parameters were significantly inhibited by varied concentrations of dihydroartemisinin and artemether. Three photosynthetic parameters examined and in each case artemether led to more significant inhibitions (p<0.01) and these inhibition effectswere more obvious with exposure time. Concretely, in 6 mg·L^-1^ artemether treatment, the Fv/Fm, ΦPSII, and ETR significantly decreased (p<0.01) after 120h exposure from 0.490, 0.516, and 17.333 to 0, respectively. The 24 mg·L^-1^ dihydroartemisinin treatment presented more significant inhibitory effects than those of 6 and 12 mg·L^-1^ treatments in the three photosynthetic parameters. After 120h cultivation, the Fv/Fm, ΦPSII, and ETR in 24 mg·L^-1^ dihydroartemisinin treatment significantly decreased from 0.491 to 0.123, from 0.509 to 0.049, and from 17.10 to 1.60, respectively. However, in 12 mg·L^-1^ dihydroartemisinin treatment the three photosynthetic parameters above decreased from 0.523 to 0.387, from 0.508 to 0.379, from 17.067 to 12.200, respectively. The results also suggested that there was a pattern of inhibitory effects on Fv/Fm, YII, and ETR that was similar in different measurement phases, which definitely illustrated the impairment of photosynthetic electron transport caused by dihydroartemisinin and artemether and the universality of the three photosynthetic parameters on the photosynthetic center photosystem II.

**Fig 5:**
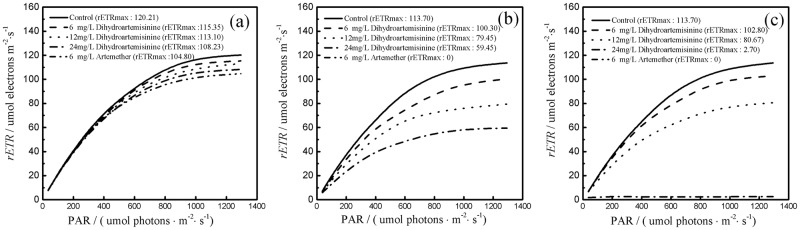
Effects of dihydroartemisinin and artemether on the relative electron transport rates (rETR) of *M*. *aeruginosa* acclimated to light irradiances ranging from 0 to 1295 μmol photons m^−2^ s^−1^ after 6h (a), 72h (b), 120h (c) treatment.

As seen in Fig 5, the results show that the relative electron transport rate increased with an increase in photosynthetically active radiation and subsequently after achieving the maximum rETR, the RLCs kept stable and level off. The RLCs of *M*. *aeruginosa* at 6, 72, 120h in dihydroartemisinin and artemether, based on rETR measurement, showed significant inhibition compared with the control.The values of rETR_max_ in artemether decreased from 104.80 at 6h to 0 μmol electrons m^-2^s^−1^ at 72 and 120 h, indicating that electron transport was completely inhibited. In the control and 6 mg·L^-1^ dihydroartemisinin, the values of rETR_max_ at 6, 72, 120h were 120.21, 113.70, 113.70 and 115.35, 100.30, 102.80 μmol electrons m^-2^s^−1^, respectively, indicating little inhibition of rETR in 6 mg·L^-1^ dihydroartemisinin treatment. However, the rETR_max_ of 24 mg·L^-1^ group at 120h was 2.70 μmol electrons m^-2^s^−1^, significantly higher than 80.67 μmol electrons m^-2^s^−1^ of the 12 mg·L^-1^ group, illustrating the inhibitory effects of 24 mg·L^-1^ treatment on rETR_max_ were more significant than those of 12 mg·L^-1^ treatment, which were similar in performance on Fv/Fm, YII, and ETR.

### Change in the extracellular APA

**Fig 6:**
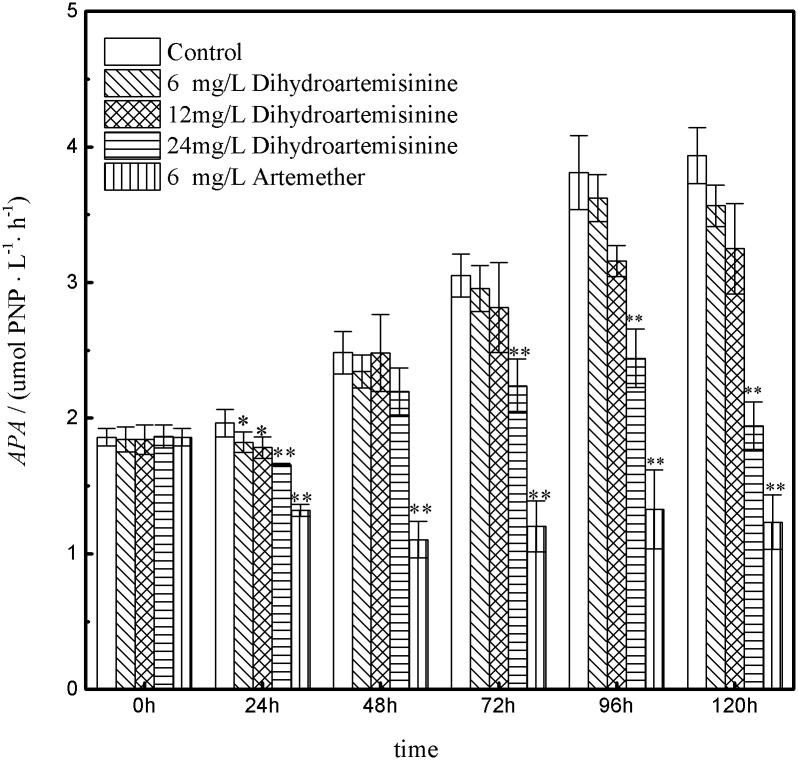
The variations of extracellular APA versus time in *M*. *aeruginosa* with dihydroartemisinin and artemether at different concentrations. *P<0.05; **P<0.01. Statistically significant differences when compared to the control.

Extracellular APA in the logarithmic phase of growth was affected differently by varying dihydroartemisinin and artemether concentrations during different exposure times (Fig 6). The total extracellular APA of the control increased from 1.86 to 3.94 μmol PNP L^−1^ h^−1^. It has been shown that both dihydroartemisinin and artemether exerted inhibitory effects on the extracellular APA compared with the control during the whole exposure time. Moreover, the results suggested the ability of inhibiting extracellular APA in artemether treatment was stronger than that of dihydroartemisinin throughout. With concentrated dihydroartemisinin, the values of extracellular APA significantly decreased (p<0.01), indicating the concentration of dihydroartemisinin played an important role in inhibiting the extracellular APA. Additionally, extracellular APA in 24 mg·L^-1^ dihydroartemisinin were significant inhibited (p<0.01) at 24, 72, 96, and 120h.

## Discussion

Biomass growth indicators such as cell number and chl-a, are routinely used as growth indicators for single-cell algae. These methods generally utilize physiological adaptations of microalgae and have a well-established eco-physiological dependence. This dependence occurs on both the spatial and temporal variability of light, nutrients, stress, and temperature conditions [20]. For this experiment, the optical density at 680 nm wavelength (OD680) was used to estimate algal biomass concentration [21]; at that wavelength, absorption and fluorescence excitation of chlorophyll can interfere with the cell scatter measurements for biomass concentration determination, and hence an estimate is obtained. Studies have found that artemether and dihydroartemisinin inhibit growth and chl-a of *M*. *aeruginosa* to different extents. Our results have demonstrated, based on economic considerations under lab conditions, the optimal dosage range for pure dihydroartemisinin to inhibit *M*. *aeruginosa* is 3.51–7.02×10^−9^ mg/cell, whereas previous studies showed 8–12×10^−9^ mg/cell was the optimal dosage for artemisininto inhibit *M*. *aeruginosa* growth well [11]. Furthermore, the decline in chl-a content of the phytoplankton species in 6 mg·L^-1^ artemether and 24 mg·L^-1^ dihydroartemisinin groups may be related to oxidative damage of chloroplast structures by reactive oxygen species [22]. However, it also demonstrated that the artemether group had greater inhibition effects than dihydroartemisinin and artemisinin at the same concentration. Apart from this outlier, these results were consistent with those of previous studies [10] detailing the inhibitory effect on the growth of *M*. *aeruginosa* by the direct addition of dihydroartemisinin and artemisinin [11]. However, the inhibition effect became weaker over time with the consumption of dihydroartemisinin and artemisinin. The mechanism responsible for this may be explained by the degradation or transformation of effective anti-algal components in allelochemical compounds [8].

Soluble proteins play key roles as enzymes in facilitating the biochemical reactions for amino acid metabolism and protein synthesis in cell metabolism. Therefore, the soluble protein level is extremely important to the growth of *M*. *aeruginosa*, which could also be reflected in the activity of cell metabolism [23–26]. The significant decrease of soluble protein content in our study indicates that artemether and dihydroartemisinin may inhibit the protein biosynthesis ability in algae, with artemether exerting more inhibitory effects. The reduction of soluble protein content may be associated with a reduction in cellular photosynthetic rates, which limits the amount of sugars/monosaccharides and other organic molecules that serve as carbon sources for protein biosynthesis [23, 26]. Protein deficiency would affect or disorder normal physiological metabolism functions, even resulting in algal death, which may be the one of algae inhibiting mechanisms.

MDA was used as a toxicity endpoint, measured after 120-h exposure. As we know, the cell membranes of algae are vulnerable to reactive oxygen species. MDA, as anoxidized product of membrane lipid peroxidation caused by reactive oxygen species, has been utilized frequently as a biomarker for vital signs of cellular oxidative damage [27–29]. Results indicate that oxidative damage caused by artemether is significantly higher than that of dihydroartemisinin, which illustrates both artemether and dihydroartemisinin can break the cell membranes and accelerate cell lysis, with *M*. *aeruginosa*more sensitive to artemether. To a certain extent, the toxicity measurement results demonstrated growth inhibition at 120h maybe be due to the oxidative impairment of membrane structures and functions caused by dihydroartemisinin and artemether, which supported the previous reports on MDA of *M*. *aeruginosa* exposed to algaecides [16, 30].

Cyanobacteria are important plankton organisms in the sea and freshwater lakes. They are photoautotrophic prokaryotes [31]. It is thought that a primary method of impairing cyanobacteria growth by algaecide is by impairing the photosynthetic system of phytoplankton [14, 32], although this mechanism has yet to be fully understood. Chlorophyll fluorescence has proven to be a useful tool for studying the behavior of PSII as it is a powerful, non-invasive, and reliable method [6, 31, 33]. As the fluorescence transient reflects the state of the primary bound plastoquinone, the secondary bound plastoquinone, the fluxes of photons, excitons, and electrons, the fast fluorescence curves were determined to show the changes of fluorescence intensity and relative variable fluorescence parameters [34]. When *M*.*aeruginosa* cells were suddenly illuminated with high intensity actinic light, all reaction centers are open. The maximal rate of photochemical reactions can now be determined precisely by measuring the initial slope [12].Why the mechanism of maximum fluorescence intensity in artemether treatment was significantly higher than in the control is not yet clearly understood.

Light absorbed by PSII is used for the reduction of the primary electron in PSII [18,35]. Fv/Fm (maximum quantum yield of primary photochemistry) is an indicator of light absorbed in photosystem II, reflecting the ratio of variable fluorescence to maximal fluorescence. The decreases Fv/Fm, ΦPSII, and ETR resulted from dihydroartemisinin and artemether inhibition of electron transport from the primary to the secondary bound plastoquinone [9]. These results showed that the acceptor side in the electron transport of PSII was a target site of dihydroartemisinin and artemether stress. The reduction in RLCs (rETR_max_) can represent the negative effects on both the energy transfer and capacity of the electron transport chain in the photosynthetic system [36]. When dosage of dihydroartemisinin and artemether increased, a decrease was observed in all photosynthetic parameters. This suggests that they have a large role in impairment of the photosynthetic system.

Our results suggest that the inactivation of PSII reaction centers and the inhibition of electron transport in the acceptor site may be responsible for the drop in photosynthesis rates after exposure to excess dihydroartemisinin and artemether. This means that, depending on the appropriate concentration, dihydroartemisinin and artemisinin can negatively affect photosynthetic rates and possibly the biosynthesis of important biomolecules in phytoplankton species [23]. Consequently, within microalgal cells, the reduction of photosynthetic rates in dihydroartemisinin and artemether treatments will induce changes in macromolecular allocation [26] and reduction in growth rates and biomass production [37].

Alkaline phosphatase plays a key role in the process of converting organic phosphorus into inorganic phosphorus and in the biogeochemical cycles of phosphorus in water [38–40], making nutrients that are more absorbable in the formation of cyanobacterial blooms. Alkaline phosphatase has often been used as an indicator of the nutritional status of phytoplankton communities in terms of phosphorus concentration [19, 41–42]. The functions of alkaline phosphatase in phytoplankton have been studied in recent years. It has been proposed that, cyanobacteria can overcome conditions of serious phosphorous depletion by alkaline phosphorous production [6, 39, 41, 43]. In the present study, we found that dihydroartemisinin and artemether could both inhibit the extracellular APA. This may indicate that the drugs reduce the ability to transformorganic phosphorus into inorganic phosphorus in the organic phosphorus utilization process of *M*. *aeruginosa*, with artemether showing more significant inhibition. In the BG11 medium, the inorganic phosphorus of dipotassium hydrogen phosphate was used as a solitaryand sufficient phosphorus source. Therefore, the effects of artemether and dihydroartemisinin on extracellular APA cannot reflect the utilization of organic phosphorus under pure culture conditions. Nevertheless, these findings on the effects of artemether and dihydroartemisinin on extracellular APA in *Microcystis aeruginosa* are helpful for applying dihydroartemisinin and artemether to inhibit overall cyanobacterial growth in field practice. The increase of extracellular APA with exposure time might be beneficial to the growth of *M*. *aeruginosa* or the release by the amounts of dead algae cells. We believe that the effects artemether and dihydroartemisinin have on extracellular APA may explain the growth inhibition in response to stress.

## Conclusion

Thus, this study showed that dihydroartemisinin and artemether have the potential to be used as algaecides, and resulted in excellent inhibition of the growth of *M*. *aeruginosa* by impairing the photosynthetic center in its photosystem II and reducing extracellular APA activity. Of the two, inhibition in artemether was greater. However, before applying dihydroartemisinin and artemetheroutside of controlled conditions, further research needs to be conducted into the feasibility and effects on whole aquatic ecosystems.
